# Calcinosis Cutis and Delayed‐Onset Myositis in a Case of Suspected Localized Scleroderma: A Diagnostic and Therapeutic Challenge

**DOI:** 10.1111/pde.70251

**Published:** 2026-04-29

**Authors:** Edoardo Marrani, Laura Gatti, Ilaria Pagnini, Teresa Oranges, Cesare Filippeschi, Gabriele Simonini

**Affiliations:** ^1^ Pediatric Rheumatology Unit, ERN‐ReCONNET Center Meyer Children’s Hospital IRCCS Florence Italy; ^2^ Neurofarba Department University of Florence Florence Italy; ^3^ Unit of Dermatology, Department of Pediatrics Meyer Children’s Hospital IRCCS Florence Italy

**Keywords:** calcinosis, idiopathic inflammatory myositis, localized scleroderma

## Abstract

A 16‐year‐old girl presenting with calcinosis cutis and localized scleroderma subsequently developed delayed‐onset idiopathic inflammatory myopathy five years after initial skin involvement. Despite the absence of typical dermatomyositis features and negative myositis‐specific antibodies, whole‐body MRI revealed extensive subclinical muscle inflammation. This rare clinical evolution highlights the importance of long‐term surveillance in pediatric autoimmune disease and supports the role of imaging in detecting early, atypical manifestations of inflammatory myopathy.

To the Editors,

1

Juvenile idiopathic inflammatory myositis (jIIM) comprises a rare group of autoimmune disorders characterized by chronic inflammation of the muscles and, often, the skin. Clinical features commonly include proximal muscle weakness, fatigue, and skin manifestations such as heliotrope rash, Gottron's papules, vasculitis, and, in some cases, cutaneous calcinosis. Cutaneous calcinosis, the deposition of calcium salts in cutaneous and subcutaneous tissues, is typically a late manifestation of juvenile dermatomyositis (JDM) and systemic sclerosis and is rarely associated with localized scleroderma [1, 2].

We present the case of a 16‐year‐old girl who developed a scleroderma‐like plaque on her right upper thigh at 11 years of age. The lesion showed hyperemic borders and patches of calcification. (Figure 1) Skin biopsy detected dermal calcinosis with associated inflammation and thickening of collagen fibers. Based on these biopsy findings, the diagnosis of localized scleroderma with secondary calcinosis cutis was considered. Systemic methotrexate (15 mg/m^2^/week) was administered for 18 months, in association with oral prednisone (2 mg/kg tapering) for the first 3 months, achieving control of disease, with no new calcium deposits. After discontinuation of methotrexate, the cutaneous disease relapsed with extension of the lesions and the appearance of new areas of calcinosis. Methotrexate and oral corticosteroids were then restarted at the same dosages.

**FIGURE 1:**
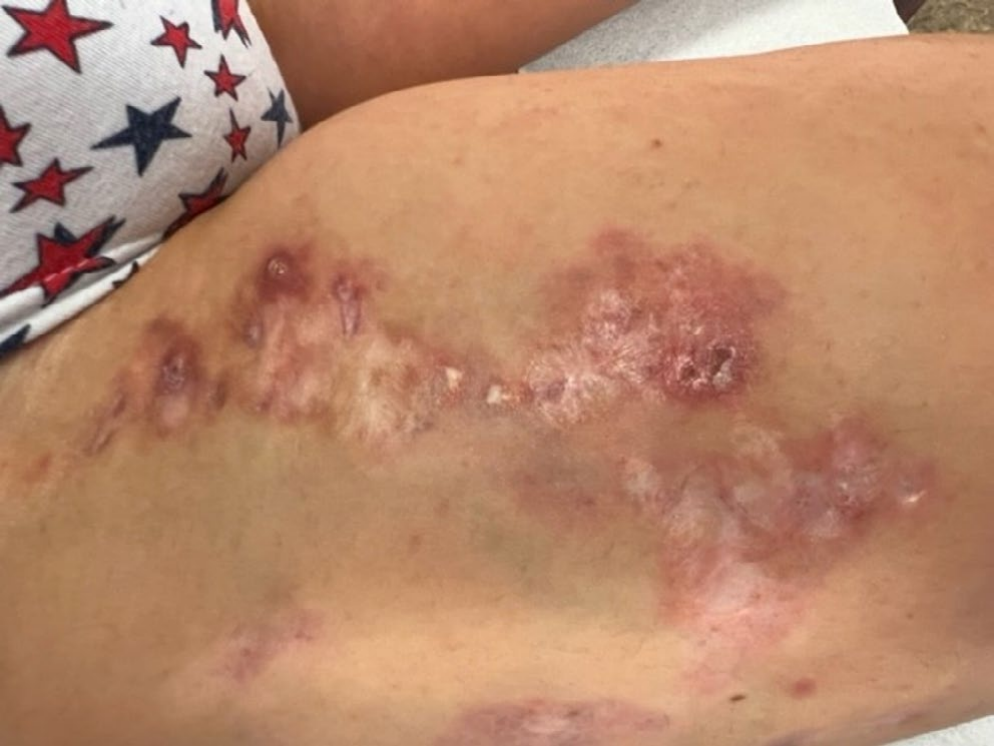
Calcinosis cutis within atrophic plaque on the right thigh, at the time of initial diagnosis, before starting systemic therapy.

Due to the atypical presentation of the lesion, thigh MRI was performed at baseline and then every 12–18 months to rule out muscle or fascial involvement as a possible alternative diagnosis of cutaneous calcinosis. At 5‐year follow‐up, at the time of weaning methotrexate dosage to 10 mg/m^2^/week, laboratory studies showed a normal aldolase, negative inflammatory markers (C‐reactive protein and erythrocyte sedimentation rate), and only mildly elevated creatine kinase (CK) level (365 UI/L, normal range 10–240 UI/L). MRI imaging was then repeated, revealing signs of inflammatory myositis: hyperintensity on T2 and STIR sequences in bilateral thigh muscles. Antinuclear antibodies were positive (1:320), but myositis‐ and systemic sclerosis‐specific autoantibodies were still negative. Whole‐body MRI identified extensive inflammation in the upper limbs and chest wall musculature (score of muscle involvement by Malattia and Damasio equal to 33/42 with score of severity 47/84) [3], with no involvement of internal organs (Figure 2). Pulmonary function tests and nail capillaroscopy were normal.

**FIGURE 2:**
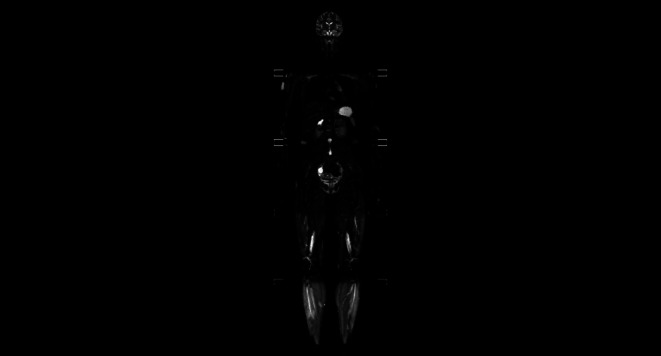
Whole‐body MRI performed 5 years after the onset of the lesion, highlighting the presence of extensive inflammation in the lower limbs.

Given the imaging evidence of myositis, treatment with baricitinib (initially 4 mg, after 1 month increased to 6 mg daily) was started together with oral methotrexate (15 mg/weekly). CK normalized after 2 months of treatment. Six months later, follow‐up MRI revealed substantial improvement, with reduced muscle involvement and only mild residual inflammation. Twelve months after starting treatment with baricitinib, the whole‐body MRI completely normalized (Figure 3).

**FIGURE 3:**
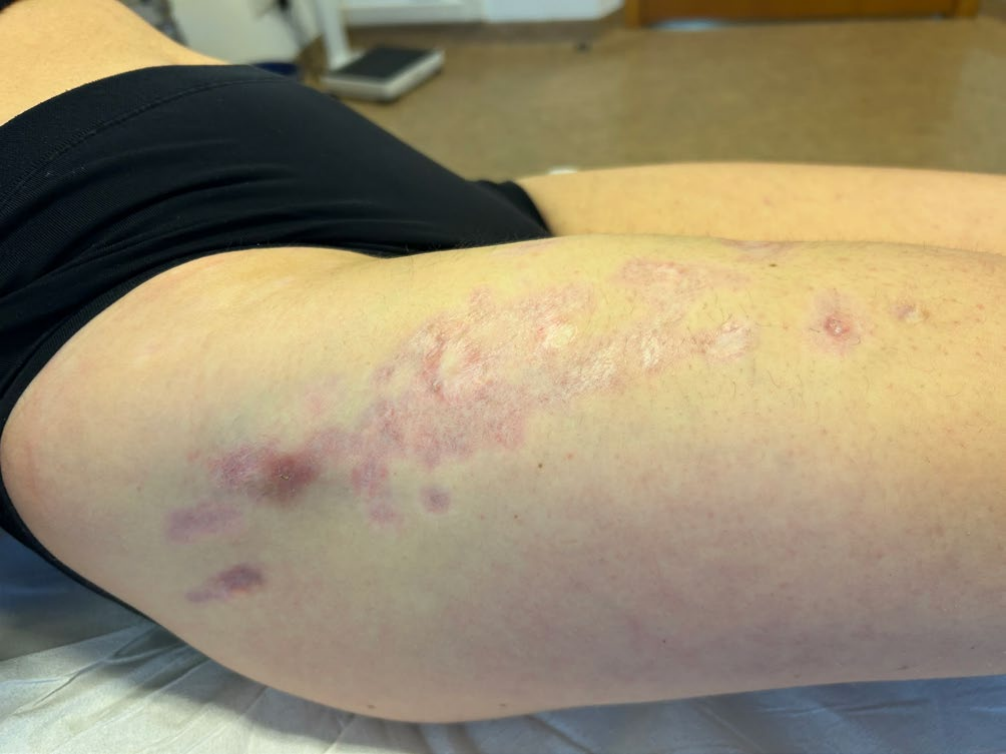
Cutaneous improvement of calcinosis after 12 months of baricitinib therapy.

Juvenile idiopathic inflammatory myopathies encompass a spectrum of phenotypes such as JDM, immune‐mediated necrotizing myopathy, and overlap myositis. Scleromyositis, a recognized subtype of overlap myositis, has clinical and serological features of both dermatomyositis and systemic sclerosis [4]. However, the overlap between localized scleroderma and inflammatory myositis is exceedingly rare. One similar case described a 19‐year‐old male who developed myositis a year after linear localized scleroderma [5].

In our patient, the presence of calcinosis cutis early in the disease course, before any evidence of muscle involvement, raises questions about the pathogenetic link between localized scleroderma and myositis. While calcinosis cutis is common in systemic connective tissue diseases, it remains exceptional in localized scleroderma, with very few reports [2]. A recent case documented a 12‐year‐old patient with calcinosis, linear morphea, lichen sclerosus, and JDM, although the causal diagnosis remained uncertain [6].

Notably, our patient never showed either cutaneous signs characteristic of dermatomyositis or expressed antibodies specific to or associated with myositis, complicating the diagnostic categorization. The possible appearance of myositis during administration of methotrexate underlines the insidious and evolving nature of autoimmune pathology in children. However, the possibility that the early presentation of calcinosis might be diagnosed as idiopathic cutaneous calcinosis cannot be excluded, although the development of later muscle involvement results even more rarely in this clinical setting [7]. Additionally, it is possible that prompt MTX use might have masked or slowed down the myositis appearance.

In conclusion, this case underscores the need for long‐term vigilance in pediatric patients with recurrent/resistant localized scleroderma, especially if associated with atypical findings such as calcinosis cutis at the disease onset. Myositis may develop later in a subtle and insidious manner, often obscured by prior autoimmune manifestations and/or ongoing treatment; therefore, a high index of suspicion is essential to detect potential subsequent manifestations.

## Author Contributions

All the authors contributed equally to the manuscript.

## Conflicts of Interest

The authors declare no conflicts of interest.

## Data Availability

Data sharing not applicable to this article as no datasets were generated or analysed during the current study.
